# A History of Epidemic Cholera, as It Appeared at the Baltimore City and County Alms-House, in the Summer of 1849, with Some Remarks on the Medical Topography and Diseases of This Region

**Published:** 1852-08

**Authors:** 


ART. VI.—*A History of Epidemic Cholera, as it appeared at the Baltimore City and County Alms-House, in the Summer of* 1849, *with some remarks on the Medical Topography and Diseases of this Region.* By TH. H. BUCKLER, Physician to the Baltimore City and County Alms-House. Baltimore: 1851.

This is a very carefully prepared paper, and embodies a large amount of useful information on a most interesting subject. We have only room for a few of the author's conclusions.

*Theories Tested.*—Before the removal of the nuisances the various cholera theories were tested as far as practicable. Saucers containing solutions of acetate of lead, nitrate of silver and other delicate reagents, were placed on the margin of the pond, and at various other points back of the north wall; and numerous strips of chemically pure paper wet with solutions of these salts were hung out at night over the different pools. Paper prepared with Schonbines' solution of iodide of potassa and starch were also used to test the presence of ozone. Duplicate experiments were instituted in the city at the same time, but without any very satisfactory results in either of the trials, the changes which occurred being nearly alike at the two places.

With the view of testing the cryptogamic and animalcular theories, plates of microscopic glass attached to threads by means of sealing wax were dipped in solutions of sugar, starch and gum acacia, and hung back of the north wall and in the cholera hospital. Other plates of glass were covered over with glycerine, remarkable for its property of remaining fluid for a long time when exposed to the air, and these, like the former, were suspended in various places about the establishment. Sugar and starch were selected because of the known tendency of vegetable germs to form on these compounds, and it was supposed if animalcula existed in the air that some of these would of necessity be caught on the moist and tenacious glycerine. These plates of glass, having been thus treated, were carefully examined by Dr. Christopher Johnston, aided by powerful lenses, but he was unable to detect the slightest trace of vegetable germs, animalculæ, or microscopic organisms of any sort.

It has been shown, in the last epidemic, that those who were least exposed to the miasm enjoyed the greatest immunity from cholera; that a partial removal of the fruitful sources of malaria was marked by a great reduction in the number of cases; and that with the entire restoration of the establishment, to a proper sanitary condition, the disease entirely ceased. It is fair then to conclude that for the existence of the local impurities, cholera would never have visited the alms-house. But it would seem that the removal of these causes of malaria, not only prevented the further spread of cholera amongst the inmates, but what is more important, that the general health of the establishment has also been greatly improved.

If the history of epidemic cholera, as it first appeared throughout this country in 1832, be compared with the invasion of 1849, it will be remarked, as one of the most striking facts connected with this disease, that it returned to every place which it had visited during the first epidemic, unless during the interval, the locality had undergone some marked change, or entire renovation. And not only so, but the last epidemic, was far more general in its distribution than the first, many places which escaped in 1832, having been visited by the disease in 1849. Moreover, since the last invasion, cholera has shown a much greater disposition to loiter about the malarious regions; this has been the case particularly at many places along the Ohio and Mississippi rivers. Even within the present month, two years and a half from the commencement of the last epidemic, there has been dreadful mortality from this disease on several plantations in the south-western States.

These remarks are not applicable to Kentucky or Tennessee, nor, we believe, to other western States. Many towns visited by cholera in 1833, have escaped the present visitation, though their condition in all respects save growth continues the same. The epidemic has been less general than it was in its former prevalence. Our observation has been, that the ordinary sources of malaria have hardly influenced it at all.

